# Can Nonfibrotic Nonalcoholic Steatohepatitis Be Effectively Identified by Supersonic Shear Imaging?

**DOI:** 10.1155/2019/2013674

**Published:** 2019-03-18

**Authors:** Jiajia Yang, Liwu Lin, Ensheng Xue, Dengke Hong, Yan Yang, Meifang Xu, Zhikui Chen

**Affiliations:** ^1^Department of Ultrasound, Fujian Medical University Union Hospital, Fuzhou, Fujian, China; ^2^Department of Vascular Surgery, Fujian Medical University Union Hospital, Fuzhou, Fujian, China; ^3^Department of Pathology, Fujian Medical University Union Hospital, Fuzhou, Fujian, China

## Abstract

Supersonic shear imaging (SSI) is a relatively new technique to measure the elasticity of target tissues based on the shear wave propagation. The aim of this study was to evaluate the value of SSI in discriminating nonfibrotic nonalcoholic steatohepatitis (NASH) from the less severe nonalcoholic fatty liver disease (NAFLD), NASH with fibrosis, and the normal liver, as well as the relationship between various NAFLD pathologic or biochemical findings and SSI liver elasticity. Rabbits with NAFLD of different degrees were subjected to SSI for liver elasticity measurement. Plasma was collected for biochemical examinations, and liver tissues were harvested for pathologic assessment. Results showed that liver elasticity of rabbits with nonfibrotic NASH was significantly different from that of rabbits with simple steatosis, borderline, NASH with fibrosis, and normal liver (*P* < 0.05) and the areas under the receiver operating characteristic curve of SSI for predicting nonfibrotic NASH and NASH with fibrosis were 0.997 and 0.967, respectively, and the optimal cutoff values were 10.17 kPa and 12.82 kPa, respectively. Multivariate analysis showed that only fibrosis and inflammation were the independent factors affecting liver elasticity of NAFLD (*P* ≤ 0.001), while inflammation, steatosis, and ballooning degeneration were all independently related to liver elasticity in rabbits without fibrosis (*P* < 0.01). In addition, alanine aminotransferase was the only biochemical factor independently related to liver elasticity (*P* ≤ 0.001). Our results indicate that SSI can effectively identify nonfibrotic NASH in rabbits based on the difference in liver elasticity and the difference is related to the various pathologic changes, including fibrosis, inflammation, steatosis, and ballooning degeneration.

## 1. Introduction

Nonalcoholic fatty liver disease (NAFLD) is a liver manifestation of metabolic syndrome and characterized by excessive accumulation of fat in hepatocytes. In recent years, the prevalence of NAFLD is increasing worldwide and it has become the leading cause of chronic liver disease [1]. The disease spectrum of NAFLD includes simple steatosis (SS), nonalcoholic steatohepatitis (NASH), and relevant fibrosis, cirrhosis, and hepatocellular carcinoma (HCC). Compared with SS, NASH has a higher risk of adverse outcomes and is more likely to cause cirrhosis, HCC, and extrahepatic complications such as diabetes and cardiovascular disease. Therefore, accurate identification and evaluation of NASH have important clinical significance [2, 3].

Liver biopsy is the gold standard for the diagnosis of NAFLD, but it is invasive and has potentially serious complications [4]. In addition, it is likely to suffer from sampling errors due to the small size of collected specimen [5]. As a result, the use of liver biopsy is limited in clinical practice. Therefore, it is more practical to develop a noninvasive technique for the diagnosis of NAFLD. Computed tomography (CT) can assess the fatty liver and its severity based on liver CT values or the differences between liver-spleen CT values, but the patient should be exposed to radiation [6]. Magnetic resonance imaging (MRI) and magnetic resonance spectroscopy (MRS) are reliable at detecting hepatic steatosis, and magnetic resonance elastography (MRE) is able to assess stiffness within the entire volume of the liver, but the high medical cost and time-consuming scanning also limit their wide application in clinical practice [7, 8]. Ultrasound examination is a safe technique and can be easily performed repeatedly. Thus, it has been a preferred tool in the diagnosis of NAFLD. However, the differentiation of NAFLD with different severities is relatively difficult based on the grayscale images in conventional ultrasonography [9].

Ultrasonic shear wave elastography techniques developed in recent years (such as transient elastography (TE), acoustic radiation force impulse (ARFI) elastography, and supersonic shear imaging (SSI)) can be used to detect the liver elasticity for the evaluation of NAFLD and have shown great potential in assisting fibrosis staging [10]. Among these techniques, SSI is a relatively new one and can remotely generate mechanical vibration sources to radiate low-frequency shear waves in tissues by using ultrasonic focused beams and monitor the propagation of resulting shear waves by using ultrafast plane wave imaging technique [11]. Compared with TE and ARFI, SSI can not only evaluate the tissue stiffness by measuring shear wave velocity but also allow the elasticity measurement in the region of interest (ROI) under the guidance of two-dimensional ultrasound images and display a real-time color map of tissue elasticity in ROI. Existing studies have shown that SSI is superior to TE and ARFI in the evaluation of liver elasticity [10, 12, 13], and its measurement is stable and reproducible [14, 15].

Available studies have confirmed that fibrosis can affect liver elasticity in the case of NAFLD, but there is no consensus about the effect of other NAFLD pathological changes (such as steatosis, ballooning degeneration, and inflammation) on the liver elasticity [15–17]. Several studies have reported that NASH can be distinguished from the NAFLD of different degrees and the normal liver by SSI, based on the significant difference in the elasticity among them, and the elasticity is considered to be related to fibrosis [15, 17]. Therefore, investigators speculate that SSI can well identify NASH with fibrosis but little is known about the value of SSI in the identification of nonfibrotic NASH [15]. In this study, SSI was employed for liver elasticity measurement in rabbits with various degrees of NAFLD, the utility of SSI in distinguishing nonfibrotic NASH from less severe NAFLD, NASH with fibrosis, and normal liver was further assessed, and the relationships between various NAFLD pathologic or biochemical findings and SSI elasticity were also explored.

## 2. Materials and Methods

### 2.1. Animal Models

A total of 40 New Zealand male rabbits (Shanghai Slack Laboratory Animal Co. Ltd.) weighing 1.80-2.23 kg were housed in isolated cages in an environment with 12 h/12 h light/dark cycle at room temperature of 22°C and relative humidity of 30%-50%, and animals were given ad libitum access to food (provided by Fujian Medical Research Institute) and water. Rabbits were housed for 1 week for accommodation to the environment and then randomly divided into four groups. Ten rabbits in the control group were fed with a standard general feed (150 g/d), and the remaining rabbits were fed with high-fat diet (HFD) (1% cholesterol, 0.5% bile salt, 5% egg yolk powder, 10% lard, and 83.5% general feed, 150 g/d, provided by Fujian Medical Research Institute) for 4 weeks (*n* = 10, HFD-4w group), 8 w (*n* = 10, HFD-8w group), or 12 w (*n* = 10, HFD-12w group) to obtain different degrees of NAFLD. At the predesigned time points, SSI measurement was performed, blood (3 ml) was collected from the ear vein, and then animals were sacrificed by injection with excessive 3% sodium pentobarbital via the ear vein. The liver tissues were collected, fixed in 10% formalin solution, and embedded in paraffin for pathologic examination.

### 2.2. Share Wave Elastography of the Liver

**Figure 1:**
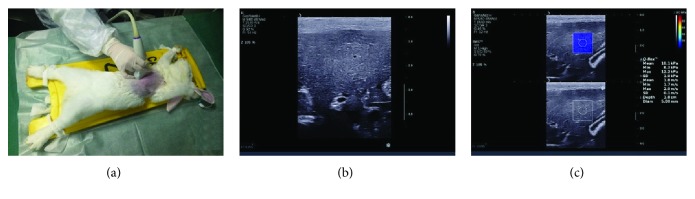
Share wave elastography of the rabbit liver. (a) After overall observation of the two-dimensional grayscale images of the rabbit liver, the line array probe (SL10-4) of an Aixplorer ultrasound imaging system was fixed lightly on the rabbit skin with the target liver lobe as the detection site. (b) Before the SWE test, the measurement section can be preselected by the two-dimensional grayscale image. (c) When the SWE mode is started, the screen displays a double frame. The two-dimensional grayscale diagram is used to guide the placement of ROI (square), and the gallbladder, biliary tract, and blood vessels shall be avoided. In the ROI is a real-time color map showing liver elasticity. After the color signal in the ROI is filled by more than 90% and stabilized, the Q-Box (circle) is activated to measure the elasticity value and the result is displayed on the screen. SWE: share wave elastography; ROI: region of interest.

SSI was performed at the predesigned time points. Before examinations, the rabbits were fasted for 12 h and then intravenously anesthetized with 3% sodium pentobarbital (30 ml/kg). The animals were fixed on the experimental table and hair on the chest and abdomen was removed. An Aixplorer ultrasound imaging system (SuperSonic Imagine, Aix-en-Provence, France) equipped with a line array probe (SL10-4) with a frequency of 4-10 MHz was used for elasticity measurements which were performed by a sonographer who had a two-year experience on elastography and were blind to the experimental design. First, the liver was observed on two-dimensional grayscale images, and then the probe was fixed lightly on the skin with the right lobe of the liver as the detection site before share wave elastography. The mean liver elasticity (Emean) was expressed in kilopascal (kPa) with a range of 0-100 kPa. The ROI of elastography was localized in the right liver lobe about 1.0-2.5 cm away from the body surface, and the gallbladder, interlobular fissure, biliary tract, and blood vessels were avoided. When the color signals filling in the ROI were over 90% and stable, the image was frozen. Then the Emean was measured in the ROI using a circular Q-Box with the diameter of 5 mm. Measurement was repeated 10 times for each rabbit, and the average was calculated as the final elasticity for further statistical analysis.  Figure 1 shows share wave elastography taken on the rabbit liver.

### 2.3. Biochemical Examinations

After 12 h fasting, blood was collected and then centrifuged to obtain plasma. The plasma samples were processed for the detection of alanine aminotransferase (ALT), aspartate aminotransferase (AST), total cholesterol (TC), triglyceride (TG), high-density lipoprotein cholesterol (HDL-C), and low-density lipoprotein cholesterol (LDL-C).

### 2.4. Pathological Examination

Paraffin-embedded tissue blocks from the right lobe of the liver where SSI was performed were sliced (7 *μ*m in thickness) and then stained with hematoxylin-eosin (HE) and Masson's trichrome (MT). A pathologist with a 5-year experience who was blind to the experiment assessed the liver sections under a CKX41 microscope (Olympus, Tokyo, Japan). Pathological assessment was performed according to the scoring system developed by Kleiner et al. [18]. Hepatic steatosis was graded according to the degree of hepatic fat infiltration: grade 0, <5%; grade 1, 5% - 33%; grade 2, 34% - 66%; and grade 3, >66%. Hepatocyte ballooning degeneration was evaluated as follows: grade 0, no degeneration; grade 1, fear balloon cells; and grade 2, many balloon cells. Inflammation was graded as follows: grade 0, none; grade 1, 1-2 foci/field; grade 2, 2-4 foci/field; and grade 3, >4 foci/field. NAFLD activity score (NAS) was the sum of scores of all pathologic characteristics ranging from 0 to 8, and the final pathological diagnosis was made on the basis of the sums: normal (NAS: 0), SS (NAS: 1-2), borderline (NAS: 3-4), and NASH (NAS: 5-8). The fibrosis was staged as follows: stage F0, none; stage F1, perisinusoidal or periportal fibrosis; stage F2, perisinusoidal and portal/periportal fibrosis; stage F3, bridging fibrosis; and stage F4, cirrhosis.

### 2.5. Statistical Analysis

Statistical analysis was performed using IBM SPSS Statistics Version 22.0 (IBM Corp., Armonk, NY, USA) and MedCalc software (MedCalc Software, Mariakerke, Belgium), and the measurement data are expressed as mean ± standard deviation (SD). One-way analysis of variance (ANOVA) followed by Bonferroni's post hoc test was used to compare the SSI measurements and the biochemical indicators among rabbits with different degrees of NAFLD. Spearman's rank correlation or Pearson correlation analysis was performed to assess the relationship between various pathological findings or biochemical indicators and SSI measurements, and multivariate linear regression analysis was used for multivariate analysis to adjust confounding factors. The receiver operating characteristic (ROC) curve was used to evaluate the diagnostic ability of SSI in predicting nonfibrotic NASH and NASH with fibrosis. The optimal cutoff value was determined according to Youden's index, and the corresponding sensitivity and specificity were computed with 95% confidence interval (CI). A value of *P* < 0.05 was statistically significant.

## 3. Results

### 3.1. Pathological Characteristics of NAFLD in Rabbits

Three rabbits from HFD groups died during the feeding period (one at 7th, 9th, and 10th week). The death was ascribed to the intolerance to HFD and relevant diarrhea. Finally, 37 rabbits were analyzed including 10 in the control group, 10 in the HFD-4w group, 9 in the HFD-8w group, and 8 in the HFD-12w group.  Table 1 shows the pathological features of NAFLD in different groups. There were 6 rabbits classified as SS in this study, 2 of which were from the control group and 4 from the HFD-4w group; 7 rabbits were classified as borderline, 6 of which were from the HFD-4w group and 1 from the HFD-8w group; 16 rabbits were classified as NASH, 6 of which had nonfibrotic NASH from the HFD-8w group, 2 had NASH with fibrosis from the HFD-8w group, and 8 had NASH with fibrosis from the HFD-12w group; the remaining 8 rabbits had normal liver from the control group. All animals were divided into the normal group (*n* = 8), SS group (*n* = 6), borderline group (*n* = 7), and NASH group (*n* = 16) according to the final pathological diagnosis. Rabbits in the NASH group were further divided into the nonfibrotic NASH group (*n* = 6) and NASH with fibrosis group (*n* = 10). The fibrosis was further staged in these animals: 26 with stage 0, 6 with stage 1, 3 with stage 2, and 2 with stage 3, but cirrhosis was not found. Among the 11 rabbits with fibrosis, one with stage 1 was from the borderline group and the other ten were from the NASH with fibrosis group.

### 3.2. Liver Elasticity in Rabbits with Various Degrees of NAFLD

**Figure 2:**
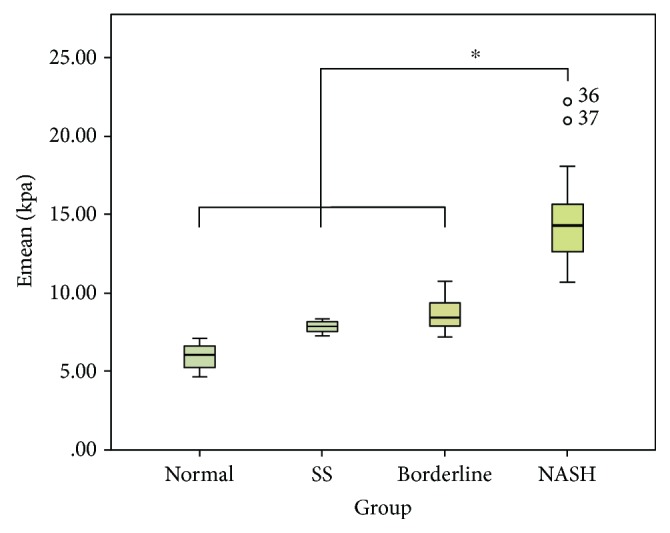
The SSI measurements of the rabbit liver with NAFLD in various severe degrees. Emean: mean elasticity value; SS: simple steatosis; NASH: nonalcoholic steatohepatitis; NAFLD: nonalcoholic fatty liver disease; SSI: supersonic shear imaging. Asterisks indicate pairs having statistically significant differences in Bonferroni's post hoc test after ANOVA test.

**Figure 3:**
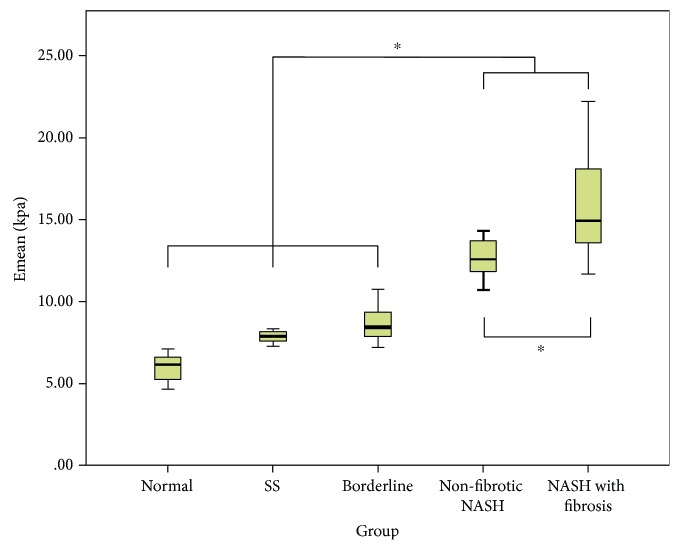
The SSI measurements of nonfibrotic NASH and NASH with fibrosis in rabbits. Emean: mean elasticity value; SS: simple steatosis; NASH: nonalcoholic steatohepatitis; SSI: supersonic shear imaging. Asterisks indicate pairs having statistically significant differences in Bonferroni's post hoc test after ANOVA test.

**Figure 4:**
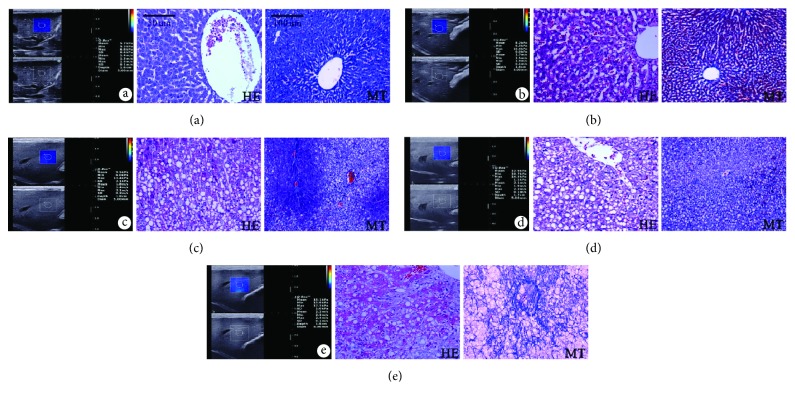
Supersonic share wave elasticity images and HE- and MT-stained liver sections in rabbit models of NAFLD. (a) From a normal rabbit liver. Share wave elasticity image shows that the Emean value was 5.7 kPa. Liver sections show normal histologic performances without steatosis, ballooning degeneration, inflammation, and fibrosis. (b) From a rabbit liver with SS. The Emean value was 8.2 kPa. Liver sections show hepatic steatosis without ballooning degeneration, inflammation, and fibrosis. (c) From a borderline rabbit liver. The Emean value was 9.5 kPa. Liver sections show dispersive hepatic steatosis with sporadic ballooning degeneration, and no significant inflammation and fibrosis can be found. (d) From a rabbit liver with NASH. The Emean value was 12.9 kPa. Liver sections show diffuse hepatic steatosis with sporadic ballooning degeneration and inflammation, without fibrosis. (e) From a rabbit liver with NASH. The Emean value was 15.1 kPa. Liver sections show dispersive hepatic steatosis with significant ballooning degeneration and hyperplasia of perisinusoidal fibrous tissue. HE: hematoxylin-eosin; MT: Masson's trichrome; NAFLD: nonalcoholic fatty liver disease; Emean: mean elasticity value; SS: simple steatosis; NASH: nonalcoholic steatohepatitis.

SSI measurements in the NASH (14.70 ± 3.24 kPa), nonfibrotic NASH (12.61 ± 1.30 kPa), and NASH with fibrosis (15.96 ± 3.44 kPa) groups were significantly higher than those in the normal (5.94 ± 0.87 kPa), SS (7.84 ± 0.40 kPa), and borderline groups (8.70 ± 1.29 kPa) (*P* < 0.05). Meanwhile, SSI measurements in the nonfibrotic NASH group were significantly lower than those in the NASH with fibrosis groups (*P* < 0.05). There was no significant difference in the liver elasticity among the normal group, SS group, and borderline group (*P* > 0.05). The SSI measurements in rabbits with various degrees of NAFLD are shown in Figures 2 and 3, and the performances of SSI and pathologic examination are presented in Figure 4.

### 3.3. Value of SSI in Predicting Nonfibrotic NASH and NASH with Fibrosis

The area under ROC curve (AUROC) of SSI for distinguishing nonfibrotic NASH from less severe NAFLD or normal liver was 0.997 (95% CI, 0.899–1.000), and the optimal cutoff value was 10.17 kPa, with the sensitivity of 100% and the specificity of 95.24%. In the differentiation of nonfibrotic NASH and NASH with fibrosis, the AUROC of SSI was 0.967 (95% CI, 0.849–0.998) and the optimal cutoff value was 12.82 kPa with the sensitivity of 90.00% and the specificity of 92.59%.

### 3.4. Correlation of Liver Elasticity with Pathologic Findings

Univariate analysis showed that the liver elasticity on SSI had a positive correlation with all NAFLD pathologic findings, including steatosis, ballooning degeneration, inflammation, and fibrosis (*P* ≤ 0.001). Multivariate linear regression analysis showed that only inflammation and fibrosis were independently related to the liver elasticity (*P* ≤ 0.001). Then univariate and multivariate analyses were performed again after excluding fibrosis and results showed that steatosis, ballooning degeneration, and inflammation were independently related to liver elasticity (*P* < 0.01). The results of univariate and multivariate analyses are shown in Tables 2 and 3.

### 3.5. Biochemical Characteristics of Rabbits with Various Degrees of NAFLD

Biochemical findings of rabbits with various degrees of NAFLD are shown in Table 4. The AST concentration in the NASH with fibrosis group was significantly higher than that in the normal group (*P* < 0.05), but there was no significant difference between the NASH with fibrosis group and the nonfibrotic NASH group (*P* > 0.05). The ALT concentration was the highest in the NASH with fibrosis group (*P* < 0.05), while concentrations of TC and HDL-C were the highest in the nonfibrotic NASH group (*P* < 0.05). Moreover, the concentrations of TC, TG, and LDL-C significantly increased in the SS and borderline groups as compared with the normal control group (*P* < 0.05).

The correlation of liver elasticity with various biochemical indicators is shown in Table 5. Univariate analysis showed that SSI measurements were significantly related to ALT, AST, TC, HDL-C, and LDL-C (*P* < 0.05), while multivariate analysis indicated that only ALT was independently related to liver elasticity (*P* ≤ 0.001).

## 4. Discussion

In available studies, mice, rats, and rabbits are frequently used to establish animal models of NAFLD [19–21]. The New Zealand male rabbits used in the present study have a large liver size, and thus, it is easy to perform ultrasound elastography of the liver. The fat intake of rabbits is relatively easy to control, leading to a high success rate of modeling. In our study, except for the 3 rabbits that died of intolerance to HFD and relevant diarrhea, the remaining rabbits in the HFD groups were all diagnosed with NAFLD of different severities.

In the disease spectrum of NAFLD, NASH has the greatest potential risk of developing into cirrhosis and even HCC. The severity of NASH and the accompanying fibrosis may directly affect the prognosis of NAFLD [22, 23]. Therefore, early accurate identification of NASH is of great significance for the timely clinical intervention and the improvement of NAFLD prognosis. The liver elasticity of the nonfibrotic NASH group in our experiment was lower than that of the NASH with fibrosis group, but significantly higher than that of the borderline, SS, and normal control groups (*P* < 0.05). These results suggest that nonfibrotic NASH can be effectively differentiated from the other degrees of NAFLD and normal liver in rabbits on the basis of SSI. ROC curve analysis showed the AUROC of SSI in predicting nonfibrotic NASH and NASH with fibrosis was 0.997 (95% CI, 0.899–1.000) and 0.967 (95% CI, 0.849–0.998), respectively and the optimal predicted cutoff values were 10.17 kPa and 12.82 kPa, respectively. However, there was no significant difference in the liver elasticity among the borderline, SS, and normal groups in the present study (*P* > 0.05), which was consistent with findings reported by Kang et al. [17].

Our study showed that liver fibrosis was a major factor that had positive influence on the liver elasticity of rabbits with NAFLD (*B* = 2.892, 95% CI 2.167-3.617, *P* ≤ 0.001). This was similar to the results of previous studies [24–26] and may be related to the increased collagens that make the liver tissues denser, finally leading to the increased elasticity on SSI (a factor positively related to the tissue density). Besides fibrosis, multivariate analysis in our study also showed that inflammation was another independent factor related to the liver elasticity of NAFLD rabbits (*B* = 1.248, 95% CI 0.519-1.977, *P* ≤ 0.001). This was consistent with that reported in previous studies [27, 28] and may be related to the inflammatory cell infiltration and increased pressure in the liver. However, our finding was different from those reported by Lee et al. [15] and Kang et al. [17]. This discrepancy might be related to the differences in experimental animals and inclusive research samples. Considering the significant influence of fibrosis and to find out the potential factors affecting liver elasticity in the NAFLD without fibrosis, we performed univariate and multivariate analyses again after excluding rabbits with fibrosis. The result showed that steatosis, ballooning degeneration, and inflammation were all independent factors positively affecting liver elasticity in rabbits without fibrosis (*P* < 0.01). The increased liver elasticity caused by ballooning degeneration is thought to be related to the increased liver tissue tension, which may be ascribed to the hepatocyte swelling [29]. Theoretically, the increased fat content in hepatic steatosis may lead to the decrease in liver tissue density and the liver elasticity decreases as well. However, opposite results were observed in our study, which was consistent with the study result of Petta et al. [30]. We speculate that this may be caused by the hepatocyte swelling due to the accumulation of lipid droplets and the increased tension of the liver tissue, which is similar with ballooning degeneration. Although the steatosis in NASH is not always more severe than that in borderline and SS livers, there is a higher degree of ballooning degeneration and inflammation which may increase the liver elasticity. Therefore, nonfibrotic NASH may be effectively distinguished by SSI from less severe NAFLD based on the higher liver elasticity.

Serum biochemical examination is another noninvasive method in evaluating NAFLD [31–33]. Among the various biochemical parameters of the liver, ALT and AST are widely used to determine the degree of hepatocyte injury; the higher the parameters are, the more serious the hepatocyte injury is, while the plasma TC, TG, HDL-C, and LDL-C mainly reflect the blood lipid and are closely related to lipid metabolism. Multivariate analysis in the present study showed that, among various biochemical indicators, ALT was the only independent factor related to liver elasticity in NAFLD rabbits (*B* = 0.169, 95% CI 0.125-0.213, *P* ≤ 0.001). ALT concentration and pathological changes of inflammation and ballooning degeneration are the indicators of liver damage; they were found in our study to be positively correlated with liver elasticity in NAFLD rabbits. Thus, we speculate that severe liver injury may increase liver elasticity on SSI. In addition, in our study, TC, TG, and LDL-C in rabbits of the SS and borderline groups were significantly higher than those in the normal control groups (*P* < 0.05), suggesting that detection of plasma TC, TG, and LDL-C may help for the evaluation of less severe NAFLD.

In this experiment, a high-fat diet was used to establish the rabbit NAFLD model and NAFLD in this model is similar to human NAFLD in terms of etiology and pathology. After 12-week high-fat diet, cirrhosis was not found in these rabbits. Different from previous studies [15, 17] in which CCl4 and other preparations were used to induce fibrosis, the NAFLD established in our study had a lower degree of fibrosis, which may be one of the reasons for the discrepancy among studies. However, the degree of fibrosis might have no influence when the correlations of liver elasticity with other pathologic characteristics were assessed after excluding rabbits with fibrosis. In addition, steatosis, ballooning degeneration, and inflammation may be present in parallel during the establishment of high-fat diet-induced NAFLD. Therefore, it is difficult to exclude other confounding pathological characteristics when the influence of each pathological characteristic on the liver elasticity is evaluated.

In conclusion, nonfibrotic NASH can be effectively identified from NAFLD of other degrees and normal liver in rabbits by SSI, based on the difference in liver elasticity. The difference in liver elasticity might be related to not only the degree of fibrosis but also the degrees of inflammation, steatosis, and ballooning degeneration in the liver with NAFLD.

## Figures and Tables

**Table 1:** Pathologic characteristics of rabbit models.

	Control group (*n* = 10)	HFD-4w group (*n* = 10)	HFD-8w group (*n* = 9)	HFD-12w group (*n* = 8)
Pathologic findings				
Steatosis grade	8/2/0/0	0/6/4/0	0/1/3/5	0/2/5/1
Ballooning grade	9/1/0	2/8/0	1/4/4	0/2/6
Inflammation grade	9/1/0/0	4/5/1/0	0/1/6/2	0/0/5/3
Fibrosis stage	10/0/0/0/0	10/0/0/0/0	6/3/0/0/0	0/3/3/2/0
NAS	8/2/0/0	0/4/6/0	0/0/1/8	0/0/0/8

HFD: high-fat diet; NAS: nonalcoholic fatty liver disease activity score.

**Table 2:** Correlation of liver elasticity values with histopathology findings in all rabbit models.

	Univariate analysis		Multivariate analysis		
	*ρ*	*P* value	*B*	95% CI	*P* value
Steatosis grade	0.695	≤0.001	0.527	-0.194-1.249	0.146
Ballooning grade	0.806	≤0.001	0.784	-0.151-1.718	0.097
Inflammation grade	0.870	≤0.001	1.248	0.519-1.977	0.001
Fibrosis stage	0.739	≤0.001	2.892	2.167-3.617	≤0.001

*ρ*: Spearman's correlation coefficient; *B*: regression coefficient. Significant *P* value is <0.05.

**Table 3:** Correlation of liver elasticity values with histopathology findings in rabbit models without fibrosis.

	Univariate analysis		Multivariate analysis		
	*ρ*	*P* value	*B*	95% CI	*P* value
Steatosis grade	0.857	≤0.001	0.926	0.254-1.598	0.009
Ballooning grade	0.708	≤0.001	1.186	0.358-2.013	0.007
Inflammation grade	0.833	≤0.001	1.089	0.426-1.751	0.003

*ρ*: Spearman's correlation coefficient; *B*: regression coefficient. Significant *P* value is <0.05.

**Table 4:** Biochemical examination results of blood plasma from rabbit models.

Pathologic diagnoses	ALT (U/l)	AST (U/l)	TC (mmol/l)	TG (mmol/l)	HDL-C (mmol/l)	LDL-C (mmol/l)
Normal	24.63 ± 7.46	30.38 ± 6.80	1.82 ± 0.48	1.34 ± 0.49	0.45 ± 0.15	1.13 ± 0.41
Simple steatosis	23.17 ± 7.89	44.50 ± 6.41	24.74 ± 8.33^a^	6.33 ± 3.45^a^	1.57 ± 1.42	25.69 ± 10.25^a^
Borderline	29.86 ± 8.73	44.14 ± 6.59	29.73 ± 8.94^a^	5.86 ± 2.35^a^	1.93 ± 1.08^a^	29.94 ± 8.18^a^
Nonfibrotic NASH	44.33 ± 6.92^ab^	58.00 ± 6.07^a^	43.00 ± 2.24^abc^	4.51 ± 1.61	4.53 ± 1.11^abc^	40.86 ± 3.27^ab^
NASH with fibrosis	67.50 ± 12.70^abcd^	70.50 ± 13.82^abc^	29.36 ± 7.64^ad^	3.95 ± 1.67	1.61 ± 0.69^d^	27.91 ± 8.09^ad^

ALT: alanine aminotransferase; AST: aspartate aminotransferase; TC: total cholesterol; TG: triglyceride; HDL-C: high-density lipoprotein cholesterol; LDL-C: low-density lipoprotein cholesterol; NASH: nonalcoholic steatohepatitis. Data are mean ± standard deviation. ^a^Compared with the normal group, *P* values < 0.05; ^b^Compared with the simple steatosis group, *P* values < 0.05; ^c^Compared with the borderline group, *P* values < 0.05; ^d^Compared with nonfibrotic NASH group, *P* values < 0.05.

**Table 5:** Correlation of liver elasticity values with biochemical findings in rabbit models.

	Univariate analysis		Multivariate analysis		
	*r*	*P* value	*B*	95% CI	*P* value
ALT	0.909	≤0.001	0.169	0.125-0.213	≤0.001
AST	0.799	≤0.001	0.008	-0.052-0.068	0.796
TC	0.568	≤0.001	-0.009	-0.121-0.103	0.874
TG	0.015	0.929	—	—	—
HDL-C	0.354	0.032	-0.045	-0.632-0.542	0.877
LDL-C	0.577	≤0.001	0.088	-0.012-0.188	0.083

ALT: alanine aminotransferase; AST: aspartate aminotransferase; TC: total cholesterol; TG: triglyceride; HDL-C: high-density lipoprotein cholesterol; LDL-C: low-density lipoprotein cholesterol; *r*: Pearson correlation coefficient; *B*: regression coefficient. Significant *P* value is <0.05.

## Data Availability

The data used to support the findings of this study are available from the corresponding author upon request.
